# New pecJ-*n* (*n* = 1, 2) Basis Sets for High-Quality Calculations of Indirect Nuclear Spin–Spin Coupling Constants Involving ^31^P and ^29^Si: The Advanced PEC Method

**DOI:** 10.3390/molecules27196145

**Published:** 2022-09-20

**Authors:** Yuriy Yu. Rusakov, Irina L. Rusakova

**Affiliations:** A. E. Favorsky Irkutsk Institute of Chemistry, Siberian Branch of the Russian Academy of Sciences, Favorsky St. 1, 664033 Irkutsk, Russia

**Keywords:** PEC method, spin–spin coupling constant, ^31^P NMR, ^29^Si NMR, phosphorus, silicon

## Abstract

In this paper, we presented new *J*-oriented basis sets, pecJ-*n* (*n* = 1, 2), for phosphorus and silicon, purposed for the high-quality correlated calculations of the NMR spin–spin coupling constants involving these nuclei. The pecJ-*n* basis sets were generated using the modified version of the property-energy consistent (PEC) method, which was introduced in our earlier paper. The modifications applied to the original PEC procedure increased the overall accuracy and robustness of the generated basis sets in relation to the diversity of electronic systems. Our new basis sets were successfully tested on a great number of spin–spin coupling constants, involving phosphorus or/and silicon, calculated within the SOPPA(CCSD) method. In general, it was found that our new pecJ-1 and pecJ-2 basis sets are very efficient, providing the overall accuracy that can be characterized by MAEs of about 3.80 and 1.98 Hz, respectively, against the benchmark data obtained with a large dyall.aae4z^+^ basis set of quadruple-ζ quality.

## 1. Introduction

Nuclear magnetic resonance (NMR) spectroscopy represents one of the most powerful tools for the chemical structure studies. For the current moment, it has become a common practice to combine high-quality quantum chemical simulation of a spectrum with the experimental NMR technique, which implies the assignment of the signals of an experimental spectrum to those of the simulated one. In this sense, quantum chemical calculations of the NMR spin–spin coupling constants and chemical shifts with high precision are of paramount importance nowadays. That is why we witness a lot of effort that is put into the development of the methodological aspects of the NMR spectra simulation, especially within past two decades [1,2].

A delicate approach is required for the calculation of the indirect nuclear spin–spin coupling constants (SSCCs), which are responsible for the splitting of resonance lines in the NMR spectra in multiplets. From one side, the efficiency of their calculation depends on the theoretical method applied. From the other side, an important issue is the quality of the basis set used. The interplay of these two factors shapes the aptness of the whole approach to the calculation of SSCCs.

Standard energy-optimized one-electron Gaussian basis sets are known to be quite inefficient for the SSCC calculations [3,4,5,6,7,8,9]. This means that one needs rather large nonspecialized basis sets to achieve the complete basis set (CBS) limit within a particular method [10]; this issue must be especially crucial for highly correlated ab initio wave-function-based methods. An efficient evaluation of the SSCCs assumes resorting to the so-called *J*-oriented basis sets [10]. The development of the *J*-oriented basis sets is not a trivial task, because each of the four Ramsey’s contributions to SSCCs, namely, Fermi-contact (FC), spin-dipolar (SD), paramagnetic spin-orbit (PSO), and diamagnetic spin-orbit (DSO), is sensitive to a particular angular region of the basis set used [8,11]. Thus, the utility of the specialized *J*-oriented basis sets consists in the fact that they reflect, to some extent, the peculiarities of the SSCCs of different types [8,11]. This obviously leads to a certain reduction of the computational cost due to their moderate sizes as compared with the nonspecialized energy-optimized basis sets that are required to attain the same accuracy.

There are already a number of specialized basis sets for the calculation of the SSCCs for the majority of popular NMR nuclei. These were obtained in different ways. The most popular and straightforward one is a consecutive augmentation of the angular spaces of standard energy-optimized basis sets with additional functions until the convergence of the total spin–spin coupling constant, or some of its dominating contributions are reached. This approach was used by Provasi, Sauer, and their colleagues to obtain now popular aug-cc-pVTZ-J basis sets for different elements: H, C–O [9,12], B, Al [7,9], F [9,13], Si [7,14,15], P, Cl [7], S [12], Sc-Zn [16], Se [17], and the 6-31G-J and 6-311G-J basis sets [3] for the density functional theory (DFT) calculations of the FC-dominating SSCCs, involving ^1^H, ^13^C, ^15^N, and ^17^O nuclei. The method of consecutive augmentation was also used by Rusakov et al. to obtain av3z-J for ^125^Te [18] and acvXz-J (X = 2, 3, 4) basis sets for ^77^Se [19], ^125^Te [19], and ^119^Sn [20] and by Helgaker et al. to obtain some *J*-oriented basis sets, namely, HX-*sun* (X = III, IV; *n* = 0–4) [21,22,23], cc-pVXZ-Cs, and cc-pVXZ-*sun* (X = D, T, Q, 5; *n* = 0–3) [11], and by Steinmann and Sauer to obtain the aug-cc-pVTZ-J basis set for the *p*-block fourth-row elements [24].

The other approaches of generating *J*-oriented basis sets involve in some way or another the optimization procedure. In particular, the search for the values of additional exponents with a variational procedure was carried out by Jensen et al., who proposed a series of famous (aug)pcJ-*n* (*n* = 0–4) basis sets [8,25,26] that are suitable for the calculation of the spin–spin coupling constants involving 1–3 row nuclei (H-Ar) with the density functional methods and are applicable to quite large systems at a favorable computational cost. Benedikt et al. also presented a series of *J*-oriented basis sets for H, He, and B-Ne, referred to as ccJ-pVXZ (X = D, T, Q, 5) [27], which were developed by the expansion of the uncontracted Dunning basis sets, cc-pVXZ(uc), with tight functions, followed by the variational procedure for the sum of absolute values of all four contributions, calculated within the coupled-cluster theory.

We also presented an efficient approach earlier, called property-energy consistent (PEC) method [28], which allows generating the basis sets for any molecular property of interest. Our method is based on the consistent optimization of all exponents using the Monte Carlo (MC) simulations [29,30,31], with respect to the property under consideration and the total molecular energy. In the mentioned work [28], we presented new pecJ-*n* (*n* = 1, 2) basis sets for high-quality calculations of the SSCCs involving 1–2 row nuclei, generated with the PEC algorithm. The PEC method was found to be capable of generating compact and efficient property-energy consistent basis sets, which provide the results of high quality, outperforming in many cases the other property-oriented basis sets of similar sizes.

In this paper, we present a continuation of our work on generating the specialized *J*-oriented basis sets with the PEC method. In this way, the goal of the present work consisted in generating accurate and computational cost-saving basis sets, purposed for the most popular NMR-active spin-1/2 nuclei of the third period, that is, ^31^P and ^29^Si. Moreover, we introduced several important modifications to our originally presented route of generating basis sets with the PEC algorithm, which significantly improved the performance of final *J*-oriented basis sets.

For the time being, there are but a few *J*-oriented basis sets for silicon and phosphorus. Namely, these are the aug-cc-pVTZ-J basis sets of Provasi and Sauer [7] and the basis sets of Jensen’s series, (aug)pcJ-*n* (*n* = 0–4) [8,25]. With these basis sets, the calculations of the silicon and phosphorus SSCCs of different types, in particular, *J*(^29^Si,^1^H) [7,14,32,33,34], *J*(^29^Si,^13^C) [35], *J*(^29^Si,^19^F) [7], *J*(^31^P,^1^H) [7,36,37,38,39], *J*(^31^P,^13^C) [40], *J*(^31^P,^17^O) [37], *J*(^31^P,^15^N) [37], *J*(^31^P,^19^F) [7], *J*(^31^P,^33^S) [37], *J*(^31^P,^77^Se) [37,41], and *J*(^31^P,^125^Te) [41], were carried out mostly within the second-order polarization propagator approach (SOPPA) [9], including its coupled cluster-modified versions [9,42], and within the density functional theory (DFT) [43]. In a scant number of papers, it was shown that specialized *J*-oriented Jensen’s and Sauer’s basis sets for silicon and phosphorus do reproduce the results obtained using much larger basis sets with favorable accuracy [7,8]. However, we suppose that there is a room left for further improvement of the accuracy of the *J*-oriented basis sets for silicon and phosphorus.

At present, one can witness that there is so small a variety of the basis sets for the calculations of SSCCs involving phosphorus or silicon that it is mandatory to fill this gap as soon as possible. Thus, we believe that the presented *J*-oriented basis sets, pecJ-*n* (*n* = 1, 2), which were obtained within a very powerful modified PEC algorithm, will prove useful in high-precision calculations of SSCCs involving phosphorus or silicon with high-quality correlated ab initio methods.

## 2. Results and Discussion

### 2.1. On the Creation of New pecJ-n (n = 1, 2) Basis Sets for Phosphorus and Silicon

The PEC method was introduced and described in detail in our previous paper [28]; therefore, we shall only briefly make mention of its main idea here. The PEC method consists in the optimization of basis sets in relation to a certain molecular property provided that the least possible total molecular energy is achieved. Exponents are randomly generated around the starting basis set via the Monte Carlo simulations. Then, generated arrays are verified whether they give the property under interest within a desired diapason or not. Of all sets that bring about the property value within the desired range, only one is selected—that one which provides the lowest energy. The main essence of the PEC algorithm, applied in this work for generating the pecJ-*n* (*n* = 1, 2) basis sets for phosphorus and silicon, was not changed, though some important modifications were introduced. The distinctions with the routine presented in ref. [28] are as follows.

First, in our previous paper [28], the generation of the *J*-oriented basis sets with the PEC algorithm was carried out using only one fitting molecule, bearing only one SSCC between eponymous nuclei. Such an approach may cause some discrepancies in the accuracy of the results obtained for different molecular systems within the same method under the same conditions [44]. Using several fitting molecules increases the robustness of the generated basis sets in relation to the diversity of the electronic systems. In this respect, for generating the basis sets purposed for each of the two considered nuclei, we employed two molecules, in each of which we selected only one SSCC of a particular type, different from that selected in another one.

Actually, it is desirable to use as many fitting molecules as possible. In the present case, given all the circumstances, among which are the large dimensions of angular spaces for the third-row elements and very costly computations within a highly correlated level of theory during the PEC optimization, we can in fact deal with no more than only two fitting molecules at once.

The second distinction between the past and present algorithms consists in the choice of the contributions to SSCCs that are to be target functions in the PEC optimization and, as a consequence, in a different way of treating the angular spaces. In a previous work [28], the basis set optimization was carried out with respect to the FC and PSO terms. At that, for the hydrogen basis set, all shells were varied, while for the case of nonhydrogen atoms, only *s*-, *d*-, and *f*-shells (with *p*-shell being fixed) were varied with respect to the FC term, and only *p*-shells were varied with respect to the PSO term. In the current case, we consider only the FC term, and all shells are being optimized entirely with respect to it. In this sense, we can say that we present new basis sets for the FC-dominating SSCCs involving phosphorus or silicon. There are also cases when the PSO term is not negligible, especially that this concerns phosphorus SSCCs [41]. The PSO term is known to be influenced mostly by the nonzero angular momentum shells, especially by the *p*-shell [18]. For the third-row elements, we chose enough large *p*- and higher angular momentum spaces to provide completeness in the particular exponential regions needed for the correct description of the PSO term. Thus, there is no need to optimize the exponents specifically for the PSO property. The SD and DSO terms are next to negligible in the vast majority of cases, and for that reason, we did not consider them as target functions.

The third distinction consists in the contraction procedure. A reasonable way to reduce the sizes of generated basis sets is to resort to a contraction scheme that can be of segmented or general type [45,46,47]. In our previous paper [28], we employed a general contraction scheme with the contraction coefficients obtained from the molecular coefficients from the self-consistent field (SCF) calculations of the simplest hydrides. This time, we performed the optimization of the contraction coefficients via the PEC algorithm. This means that the optimization of the contraction coefficients was carried out sequentially for each shell (one after another while keeping the previous shells unchanged) to minimize the absolute contraction error while ensuring that the lowest possible molecular energy was achieved. The same approach of obtaining the contraction coefficients was successfully applied by us when generating the contracted pecS-*n* basis sets for the NMR chemical shifts of 1–2 row nuclei [48]. In fact, we believe that fine proportions of primitives, settled via the PEC optimization procedure this time, will give rise to more effective contracted functions than that which could have been obtained from a widely used SCF approach.

Going into details, we used two sets of fitting molecules, namely, PH_3_ and HCP, and SiH_4_ and HSiCH, for generating basis sets for phosphorus and silicon, respectively. Thus, the PEC optimization procedure was performed in relation to the FC contributions (*J*_FC_) to a couple of one-bond SSCCs involving phosphorus, namely, ^1^*J*(^31^P,^1^H) in PH_3_ and ^1^*J*(^31^P,^13^C) in HCP, and to a couple of one-bond SSCCs involving silicon, namely, ^1^*J*(^29^Si,^1^H) in SiH_4_ and ^1^*J*(^29^Si,^13^C) in HSiCH. Thus, our extended PEC method performs simultaneous optimization of all exponents minimizing the average absolute deviation of the *J*_FC_ terms from their “ideal” values, which are the best achievable values of the FC contributions, close to a CBS limit ($J_{i}^{ideal}$):

(1) $$\bar{\Delta }=\frac{1}{2}\overset{2}{\underset{i=1}{\sum }}\left|{\tilde{J}}_{i}-J_{i}^{ideal}\right|\to min$$

This is performed under the energetic constraint $\sum_{n=1}^{2}{\tilde{E}}_{n}\to min$, which guarantees that the least possible total molecular energy of two molecules is achieved. The energy tolerance threshold was set to 10^−4^ Hartree.

The calculations of *J*_FC_ were performed using the SOPPA(CCSD) approach [9,42], while the molecular energies were calculated at the CCSD [49,50] level of theory. The “ideal” values were also obtained at the SOPPA(CCSD) level of theory, using an artificially extended dyall.aae4z basis set, designated here as dyall.aae4z^+^. To obtain the dyall.aae4z^+^ basis set, we augmented the original dyall.aae4z basis set with several tight *s*-type functions depending on the element, following an even-tempered manner [51]. The details on how the dyall.aae4z basis set was extended are given in Table 1.

The convergence of ^1^*J*(^1^H,^1^H) in H_2_ and ^1^*J*(^13^C,^13^C) in C_2_H_2_ upon sequential augmentation of the dyall.aae4z basis set with both tight and diffuse exponents in all shells was preliminarily investigated by us at the SOPPA(CCSD) level. It was found that three and two additional tight *s*-type functions for the hydrogen and carbon atoms, respectively, are sufficient to achieve the convergence. We also carried out the same investigation for all one-bond SSCCs in the fitting molecules that were mentioned above, namely, ^1^*J*(^31^P,^1^H) in PH_3_, ^1^*J*(^31^P,^13^C) in HCP, ^1^*J*(^29^Si,^1^H) in SiH_4_, and ^1^*J*(^29^Si,^13^C) and ^1^*J*(^29^Si,^1^H) in HSiCH. At that, we used the dyall.aae4z^+^ basis on hydrogens and carbons in these molecules. First, the original dyall.aae4z basis set was set on phosphorus and silicon; then, it was sequentially augmented by adding one-to-four tight *s*-type functions. At that stage, we came to the conclusion that one tight *s*-function is enough to reach the convergence of all the considered SSCCs with phosphorus and silicon. To be more precise, upon adding one tight *s*-function to the *s*-space of the dyall.aae4z basis set for phosphorus, the biggest change was observed for ^1^*J*(^31^P,^1^H) in the PH_3_ molecule, resulting in a hardly noticeable increase of the constant by about 0.125 Hz in relation to the total value of ca. 192 Hz obtained with the original dyall.aae4z basis set. For the silicon basis set, the largest change was demonstrated by the ^1^*J*(^29^Si,^1^H) SSCC in HSiCH, where the addition of one tight *s*-function to the *s*-space of the dyall.aae4z basis set caused the decrease in the original value by about 0.317 Hz. Further expansion of the *s*-spaces of the dyall.aae4z basis set for phosphorus and silicon had no effect on all considered SSCCs. Thus, we stopped at adding only one *s*-function to the *s*-space of the dyall.aae4z basis set for phosphorus and silicon. Let us call it here dyall.aae4z + 1*s*_tight_.

Then, following the line of sequential expansion, we took a dyall.aae4z + 1*s*_tight_ basis set and added to it one-to-three diffuse *s*-type functions. No effect on the SSCCs arose from that action. After that, we considered the extension of the *p*-shell of the dyall.aae4z + 1*s*_tight_ basis set towards both ends, and again, there was no noticeable effect found. Continuing the saturation of the dyall.aae4z + 1*s*_tight_ basis set up to the *g*-shell, we found that all nonzero angular momentum shells of the dyall.aae4z basis set are already complete enough to provide the converged values of the SSCCs involving phosphorus and silicon, so that these shells do not need to be extended. In that way, the dyall.aae4z^+^ basis set for silicon and phosphorus is the dyall.aae4z basis set that has been augmented with only one tight *s*-type function, that is, “dyall.aae4z^+^ = dyall.aae4z + 1*s*_tight_”.

For the sake of comparison, it is worth mentioning here that the largest available basis set of the famous Dunning series, the aug-cc-pV6Z basis set [52], for phosphorus and silicon, taken in the uncontracted form (22*s*,15*p*, 6*d*, 5*f*, 4*g*, 3*h*, 2*i*), consists of only 22 *s*-type functions with the heaviest exponent *ζ* being equal to 5.384 × 10^6^ for P and 4.465 × 10^6^ for Si. These are rather low values as compared with the heaviest 25th *s*-type exponents of the original dyall.aae4z basis set, which are 5.5229311 × 10^7^ and 4.85467489 × 10^7^ for P and Si, accordingly. Another striking difference between the dyall.aae4z and aug-cc-pV6Z basis sets lies in the *d*-shells. The uncontracted aug-cc-pV6Z has only six *d*-functions with the heaviest exponent of only 4.3008 for P and 3.2386 for Si. At the same time, the dyall.aae4z basis set contains as much as 10 *d*-functions with the tightest *d*-exponents equal to 2.98952112 × 10^2^ for P and 2.59736848 × 10^2^ for Si. These figures are two orders of magnitude more than that of the aug-cc-pV6Z basis set. There is no need to go deeper in comparative analysis of the rest of the shells to make a conclusion that the largest available energetically optimized aug-cc-pV6Z basis set for phosphorus and silicon is not as complete as the dyall.aae4z basis set in the important exponential regions, in particular, in the *s*- and *d*-shells. Thus, out of these two basis sets, the dyall.aae4z basis set is obviously the better choice to start with the saturation in order to obtain the converged “ideal” values.

Thus, the “ideal” values of FC contributions to ^1^*J*(^31^P,^1^H) in PH_3_, ^1^*J*(^31^P,^13^C) in HCP, ^1^*J*(^29^Si,^1^H) in SiH_4_, and ^1^*J*(^29^Si,^13^C) in HSiCH, calculated with the dyall.aae4z^+^ basis set, which were inputted into the PEC optimization algorithm, are as follows: 186.19, 17.12, −191.37, and −336.42 Hz, respectively.

To prepare trial starting basis sets for the PEC algorithm, we took Dunning basis sets for phosphorus and silicon of double- and triple-*ζ* quality in uncontracted form (uc), cc-pVDZ(uc) (12*s*, 8*p*, 1*d*) and cc-pVTZ(uc) (15*s*, 9*p*, 2*d*,1*f*) [53], and extended them in an even-tempered manner with two *s*- and *d*-functions in both cases. Note that the compositions of uncontracted pecJ-*n* basis sets are the same as that of the corresponding trial basis sets, because the compositions are not varied throughout the PEC optimization procedure. Thus, the compositions of the pecJ-*n* basis sets are as follows: (14*s*, 8*p*, 3*d*) for the pecJ-1 basis set and (17*s*, 9*p*, 4*d*, 1*f*) for the pecJ-2 basis set. The reason why we extended the *s*- and *d*-shells of the Dunning basis sets precisely with two functions is that it is this number of additional functions that give minimally necessary dimensions of *s*- and *d*-shells for the PEC algorithm. Less functions give the higher least possible mean absolute deviation and higher least total molecular energy. More functions are surplus as the they give the same least possible mean absolute deviation and least total molecular energy as the compositions with 14/17 *s*- and 3/4 *d*-functions for *n* = 1/2. We also did not change the dimension of the *p*-shell, because the FC term was found to be insensitive to the modifications of the *p*-shell for the atoms of the second row and beyond [7,19]. In that way, trial basis sets, ready for optimization, were set on phosphorus and silicon atoms, while on the hydrogens and carbons, we used previously developed pecJ-1 or pecJ-2 basis sets [28], depending on the level of the basis set under optimization.

The sizes of the uncontracted basis sets, pecJ-*n*(uc), obtained with the PEC method, were significantly reduced by means of employing the general contraction scheme. As was mentioned above, we applied the PEC algorithm to obtain the contraction coefficients. In this problem, the main goal was to minimize the contraction error, providing the least possible molecular energy. In more detail, to get the contraction coefficients for the phosphorus pecJ-*n* basis sets, we minimized (via the PEC method) the absolute differences between the values of ^1^*J*(^31^P,^1^H) in the PH_3_ molecule obtained with the contracted and uncontracted pecJ-*n* basis sets, with respect to the contraction coefficients. The same was performed for the silicon basis sets, where the contraction errors were minimized for ^1^*J*(^29^Si,^1^H) in the SiH_4_ molecule. It is worth mentioning here that the resulting compositions of the contracted basis sets were found to be minimally necessary in the sense of providing zero contraction error for the chosen ^1^*J*(^31^P,^1^H) and ^1^*J*(^29^Si,^1^H) SSCCs and the least molecular energy. This means that if a deeper contraction scheme is applied, the larger contraction errors and higher minimal molecular energies are occurred in the end of the PEC procedure, while less succinct compositions lead to no improvement. The resulting contraction schemes are presented in Table 2.

The exponents and contraction coefficients for our new pecJ-1 and pecJ-2 basis sets for silicon and phosphorus are presented in the Supporting Information file in Dalton and CFOUR formats.

### 2.2. The Performance of New pecJ-n (n = 1, 2) Basis Sets

In the first place, we examined the results obtained with the contracted basis sets, pecJ-*n* (*n* = 1, 2), against the “ideal” benchmark values obtained with the dyall.aae4z+ basis set on the example of 62 SSCCs involving silicon or/and phosphorus in 20 molecules. These calculations were performed at the SOPPA(CCSD) level of theory. In fact, the comparison of the theoretical results obtained using the designed basis sets with those obtained with the best physically achievable basis sets for the time period is a common practice [8,48]. This is justified when one cannot totally rely upon the accuracy of the vibrational, solvent, and relativistic corrections required for the proper comparison with the experiment. The issue especially concerns the calculation of the relativistic corrections that represent for now the most disturbing factor of uncertainty, responsible for bringing about undesirable unaccounted errors, which can unpredictably affect the analysis of the basis set performance.

The performance of our new pecJ-*n* (*n* = 1, 2) basis sets was compared with that of Jensen’s basis sets, pcJ-*n* (*n* = 1, 2), and Sauer’s basis set, aug-cc-pVTZ-J. In the calculations of the P-H SSCC in the OPH_3_ molecule with the pecJ-*n* basis sets, we used pcJ-*n* basis sets on the oxygen atom due to the absence of the pecJ-*n* basis sets for this element. The results are presented in Table 3.

**Figure 1:**
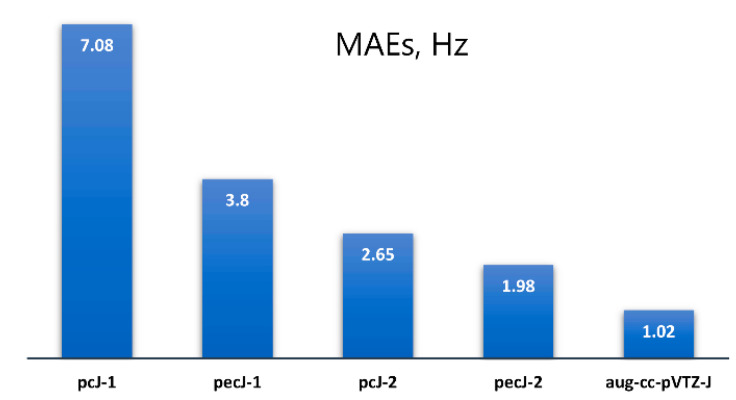
The MAEs evaluated for the SSCCs with phosphorus or/and silicon in molecules **1–20** calculated at the SOPPA(CCSD) level with pcJ-1, pcJ-2, pecJ-1, pecJ-2, and aug-cc-pVTZ-J basis sets against the benchmark data, obtained with the dyall.aae4z^+^ basis set.

Mean absolute errors (MAEs) were calculated for the results obtained with all considered basis sets against the dyall.aae4z^+^ data. These figures are as follows: 3.80, 1.98, 7.08, 2.65, and 1.02 Hz for the pecJ-1, pecJ-2, pcJ-1, pcJ-2, and aug-cc-pVTZ-J basis sets correspondingly (see Figure 1).

It can be concluded that, in general, our first- and second-level basis sets, pecJ-1 and pecJ-2, demonstrate a better accuracy as compared with the pcJ-1 and pcJ-2 basis sets, respectively.

Going into detail, it can be said that the pecJ-1 basis set provides the accuracy, which is noticeably higher than that reached with the pcJ-1 basis set and is somewhat lower than that of the pcJ-2 basis set. In terms of accuracy, one can place the pecJ-1 basis set somewhere in between the pcJ-1 and pcJ-2 basis sets, essentially closer to the latter. Pertaining to the sizes of the contracted basis sets under comparison (see Table 4), the pecJ-1 basis set is practically of the same size as the pcJ-1 basis set for the 1–2 row elements, while for the phosphorus and silicon, it is only 7 functions larger than the pcJ-1 basis set and as much as 16 functions smaller than the pcJ-2 basis set. It is also worth noting that the uncontracted pecJ-1(uc) basis set exceeds the uncontracted pcJ-1(uc) basis set in size by only 1 function for the first- and second-row elements and by only 3 functions for the third-row elements.

Thus, on the performance of the pecJ-1 basis set, we can resume that it provides the accuracy, which is significantly better than that of the pcJ-1 basis set, approaching that of the pcJ-2 basis set. At the same time, the pecJ-1 basis set is close in size to the pcJ-1 basis set while being essentially smaller than the pcJ-2 basis set.

The accuracy of our second-level basis set, pecJ-2, occurred to be somewhere in the middle between that provided by the pcJ-2 and aug-cc-pVTZ-J basis sets. At that, the size of the pecJ-2 basis set is smaller than that of the pcJ-2 basis set for the 1–2 row elements and is only one function larger for the third-row elements. The aug-cc-pVTZ-J basis set is slightly larger than the pecJ-2 basis set for the 1–2 row elements, and is noticeably larger than the pecJ-2 basis set for phosphorus and silicon. Therefore, it is not surprising that the accuracy, provided by the aug-cc-pVTZ-J basis set, occurred to be slightly higher than that provided by the pecJ-2 basis set.

In general, by these calculations, we confirm one more time that our PEC method is capable of generating small and very efficient property-energy consistent basis sets that provide results of high quality, comparable to or even better than that provided by the other property-oriented basis sets of similar sizes.

We also tested the performance of our basis sets against the experimental data on the example of five molecules, namely, phosphabenzene (**1**), phosphaethyne (**2**), phosphane (**8**), fluorosilane (**16**), and silane (**17**). Taking into account what was said above about the factors of uncertainty originating from the vibrational, solvent, and relativistic corrections, and from the basic level of theory per se, we carried out all calculations at the highest achievable levels of theory that we can afford for now.

For this purpose, we calculated the basic values at the CCSD level of theory using the pecJ-1 and pecJ-2 basis sets. The zero-point vibrational corrections (ZPVC) to SSCCs were evaluated at the SOPPA level for molecules **1** and **16** and at the CCSD level for molecules **2**, **8**, and **17**, within the vibrational second-order perturbation theory (VPT2) [23,54]. The ZPVC corrections were evaluated while taking into account both harmonic and anharmonic contributions. The pecJ-1 basis set was used in all vibrational calculations due to the extremely large computational cost of the problem. Solvent corrections were estimated at the DFT-PBE0 [55,56] level of theory within the polarizable continuum model using the integral equation formalism (IEF-PCM) [57,58] and the same basis sets as in the calculations of the basic values. Relativistic corrections were evaluated at the DFT-PBE0 level of theory as the differences between the four-component relativistic values and the nonrelativistic values. In both the relativistic and nonrelativistic calculations, the dyall.acv4z basis set [59] was used. In the four-component relativistic calculations, we applied the restricted kinetic balance condition [60,61,62] to generate the small component spinor basis space from the large spinor basis components. The results are presented in Table 5.

The results presented in Table 5 show that our basis sets, applied in the CCSD calculations by taking into account vibrational, solvent, and relativistic corrections, gave a rather good agreement with the experimental data. A harsh checkout of the efficacy of our basis sets was provided by molecules **1**, **2**, and **16**, which are computationally challenging systems possessing an intricate electronic structure.

In particular, the final theoretical value of the one-bond P-C SSCC in the unique trivalent phosphorus compound **2**, obtained with the pecJ-1 basis set, occurred to be very encouraging, for the deviation of the total theoretical value from the experimental data is only 0.64 Hz. A challenging aromatic system of compound **1** can also be said to be described well in the computation of its SSCCs within a given methodology involving our basis sets. The calculation of Si-F SSCCs per se, such as ^1^*J*(Si,F) in molecule **16**, represents a highly arduous task due to the involvement of the fluorine nucleus, requiring all state-of-the-art approaches of modern computational NMR. For example, as it follows from the calculations of ^1^*J*(Si,F) in the SiF_4_ molecule by Provasi and Sauer [7], the difference between the values obtained at the DFT-B3LYP [67,68] and SOPPA(CCSD) levels of theory amounts to approximately 160 Hz with the latter being rather closer to the experimental value (191.4 against 178.0 Hz). This speaks of an utmost importance of the proper treating of the correlation effects when calculating the Si-F SSCCs. For ^1^*J*(Si,F) in molecule **16**, we obtained surprisingly good results, with the theoretical “pecJ-2 value” deviating from the experimental datum approximately by only 3%.

Overall, incorporated in the methodology based on the CCSD calculations, our basis sets perform well for such challenging electronic systems as that of compounds **1**, **2**, and **16**. However, it is evident that the CCSD method is not a sufficiently correlated approach for these three systems. In such cases as these, it is likely that one should go beyond the configuration space of double excitations and use more advanced approaches, such as CCSDT [69,70] or higher. Moreover, given the total significance of the vibrational, solvent, and relativistic corrections to SSCCs in systems **1**, **2**, and **16**, we suspect them to play a non-negligible role in the observed disagreement of the theoretical values with the experiment. This especially pertains to the corrections obtained at the DFT level of theory, which might be of small use for challenging compounds **1**, **2**, and **16**. The calculations of SSCCs in the PH_3_ (**8**) and SiH_4_ (**17**) molecules gave very good results (see Table 5).

## 3. Computational Details

All geometry optimizations were performed using the CCSD method without taking into account media effects (gas phase), within the CFOUR program [71]. At that, we used the aug-cc-pV5Z basis set [53,72,73] on all atoms when obtaining the equilibrium geometries for our fitting molecules (PH_3_, HCP, SiH_4_, and HSiCH). For the rest of the molecules, we used the aug-cc-pVQZ basis on all atoms [53,72,73]. All obtained equilibrium geometries are presented in the Supporting Information file.

All calculations of SSCCs (including that of the vibrationally averaged values), performed at the SOPPA(CCSD) or CCSD levels of theory, were carried out in the Dalton [74] or CFOUR programs, respectively. Solvent corrections were calculated at the nonrelativistic DFT-PBE0 level of theory using the IEF-PCM model within the Dalton program. Relativistic values were calculated at the four-component DFT-PBE0 level of theory, within the DIRAC program [75]. Nonrelativistic counterparts, used for the evaluation of the relativistic corrections, were calculated at the DFT-PBE0 level, within the Dalton program.

For the basis set optimization, we used a modified PEC algorithm, which was coded by us within the Python 3.097 media [76].

## 4. Concluding Remarks

In this paper, we presented new *J*-oriented basis sets, pecJ-*n* (*n* = 1, 2), for phosphorus and silicon, which we expect to be quite efficient in the high-quality correlated calculations of the NMR spin–spin coupling constants involving these nuclei. The pecJ-*n* basis sets were generated via the property-energy consistent (PEC) method, developed by us in an earlier work. This time, we applied several important modifications to the original PEC procedure, which improved the overall accuracy and robustness of the generated basis sets in relation to the diversity of electronic systems. In the optimization procedure, to calculate the SSCCs, we resorted to the SOPPA(CCSD) method, which presents one of the most accurate correlated nonempirical methods, currently applied for the calculations of SSCCs of different types.

Our new basis sets were successfully tested on a great number of SSCCs, involving phosphorus or/and silicon, calculated within the SOPPA(CCSD) method. The accuracy of the results, obtained with our basis sets, was assessed against the benchmark data, calculated using a very large dyall.aae4z^+^ basis set, which was shown to provide the converged values of the SSCCs with phosphorus or silicon. In general, it was found that our new pecJ-1 and pecJ-2 basis sets are very efficient, providing the overall accuracy that can be characterized by MAEs of about 3.80 and 1.98 Hz, respectively. In that way, the accuracy of the pecJ-1 basis set is essentially larger than that of the pcJ-1 basis set and is close to the accuracy of the pcJ-2 basis set. At the same time, our second-level basis set, pecJ-2, demonstrated very good performance, providing the accuracy in the middle between that of the pcJ-2 and aug-cc-pVTZ-J basis sets.

## Figures and Tables

**Table 1:** Extension of the dyall.aae4z basis set to dyall.aae4z^+^.

Original dyall.aae4z	dyall.aae4z^+^	Additional *ζ*_i_
H: (12*s*, 4*p*, 3*d*, 2*f*)	+3*s*	*ζ*_1_ = 1.42009326 × 10^6^ *ζ*_2_ = 2.05353505 × 10^5^ *ζ*_3_ = 2.96952766 × 10^4^
C: (19*s*, 11*p*, 6*d*, 4*f*, 2*g*)	+2*s*	*ζ*_1_ = 6.64165270 × 10^7^ *ζ*_2_ = 9.15768223 × 10^6^
Si, P: (25*s*, 15*p*, 10*d*, 7*f*, 4*g*)	+1*s*	Si: *ζ*_1_ = 3.37124315 × 10^8^ P: *ζ*_1_ = 3.68247433 × 10^8^

**Table 2:** Contraction schemes of the pecJ-*n* basis sets for P and Si.

Basis Set	Simple Contraction Scheme	Extended Contraction Scheme	*N_c_*/*N_uc_*
pecJ-1	(14*s*, 8*p*, 3*d*|8*s*, 5*p*, 3*d*)	8*s*: (8, 8, 1, 1, 1, 1, 1, 1) 5*p*: (5, 5, 1, 1, 1) 3*d*: (1, 1, 1)	38/53
pecJ-2	(17*s*, 9*p*, 4*d*, 1*f*|10*s*, 6*p*, 4*d*, 1*f*)	10*s*: (9, 9, 1, 1, 1, 1, 1, 1, 1, 1) 6*p*: (5, 5, 1, 1, 1, 1) 4*d*: (1, 1, 1, 1) 1*f*: (1)	55/71

**Table 3:** SSCCs (in Hz) calculated at the SOPPA(CCSD) level with dyall.aae4z^+^, pecJ-*n* (*n* = 1, 2), pcJ-*n* (*n* = 1, 2), and aug-cc-pVTZ-J basis sets.

#	Molecule	SSCC ^1^	dyall.aae4z^+ 2^	pecJ-1	pecJ-2	pcJ-1	pcJ-2	aug-cc-pVTZ-J
**1**	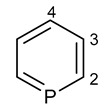 Phosphabenzene	^1^*J*(P,C_2_)	−46.27	−37.68	−43.13	−29.57	−42.41	−42.82
		^2^*J*(P,C_3_)	−19.88	−19.72	−19.87	−20.23	−20.16	−19.99
		^3^*J*(P,C_4_)	29.52	28.78	29.27	28.46	29.44	28.40
		^2^*J*(P,H_2_)	32.96	30.81	32.34	30.81	32.05	33.28
		^3^*J*(P,H_3_)	9.49	9.61	9.27	9.66	9.40	9.49
		^4^*J*(P,H_4_)	−7.01	−7.13	−6.74	−6.89	−7.08	−6.84
**2**	P≡CH Phosphaethyne	^1^*J*(P,C)	78.11	74.25	76.46	90.63	73.94	76.83
		^2^*J*(P,H)	53.72	50.97	53.03	51.87	52.34	52.59
**3**	H_2_N–PH_2_ Phosphanamine	^1^*J*(P,N)	−3.73	0.93	−2.65	1.71	−2.50	−3.85
		^1^*J*(P,H)	191.34	188.90	190.64	189.73	191.82	191.61
		^2^*J*(P,H)	11.81	10.91	11.49	11.98	11.35	11.61
**4**	O=PH_3_ Phosphine oxide	^1^*J*(P,H)	445.05	441.47	441.86	439.92	446.41	445.73
**5**	PF_3_ Phosphorus trifluoride	^1^*J*(P, F)	−1409.57	−1430.81	−1381.37	−1337.65	−1382.72	−1402.76
**6**	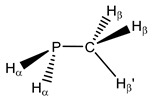 Methylphosphane	^1^*J*(P,C)	0.00	5.84	2.00	10.03	3.43	0.73
		^1^*J*(P,H_α_)	195.19	194.72	194.30	190.91	195.49	195.81
		^2^*J*(P,H_β_)	7.56	6.24	6.94	6.65	6.94	7.26
		^2^*J*(P,H_β’_)	−8.81	−9.08	−9.07	−9.05	−9.16	−9.24
**7**	H_2_P–F Fluorophosphane	^1^*J*(P,F)	−798.09	−821.89	−790.47	−736.60	−782.17	−810.23
		^1^*J*(P,H)	191.59	190.72	192.66	193.93	192.24	191.96
**8**	PH_3_ Phosphane	^1^*J*(P,H)	190.63	190.52	190.46	184.99	190.56	191.18
**9**	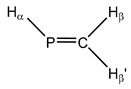 Methylenephosphane	^1^*J*(P,C)	−25.56	−15.30	−22.50	−7.66	−21.78	−21.93
		^1^*J*(P,H_α_)	131.63	126.48	132.19	126.73	129.69	132.85
		^2^*J*(P,H_β_)	−33.11	−31.93	−32.87	−31.12	−33.73	−33.84
		^2^*J*(P,H_β’_)	24.18	21.36	23.02	23.38	22.95	24.04
**10**	F_2_P–H Difluorophosphane	^1^*J*(P,H)	201.52	198.47	202.60	209.59	202.51	201.56
		^1^*J*(P,F)	−1152.93	−1168.95	−1133.17	−1074.49	−1124.99	−1152.84
**11**	H_2_C=SiH_2_ Methylenesilane	^1^*J*(Si,C)	−130.07	−132.52	−131.53	−137.73	−132.39	−132.47
		^1^*J*(Si,H)	−241.84	−241.10	−240.39	−244.05	−244.16	−241.78
		^2^*J*(Si,H)	4.85	7.40	5.58	8.28	5.91	5.21
**12**	CH≡SiH Methylidynesilane	^1^*J*(Si,C)	−378.85	−375.00	−377.57	−381.61	−380.36	−382.16
		^1^*J*(Si,H)	−445.90	−445.84	−443.48	−452.96	−450.82	−446.72
		^2^*J*(Si,H)	−112.00	−104.22	−109.70	−98.79	−108.23	−111.40
**13**	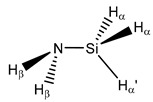 Silanamine	^1^*J*(Si,N)	−15.26	−16.17	−15.61	−16.56	−15.96	−15.67
		^1^*J*(Si,H_α_)	−199.09	−197.59	−197.50	−199.06	−200.47	−199.21
		^1^*J*(Si,H_α’_)	−188.85	−186.41	−187.08	−186.78	−189.67	−188.83
		^2^*J*(Si,H_β_)	−1.11	−0.80	−1.04	−1.05	−1.10	−1.04
**14**	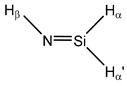 Silanimine	^1^*J*(Si,N)	−4.53	−5.37	−4.73	−6.39	−4.81	−5.02
		^1^*J*(Si,H_α_)	−214.71	−208.67	−211.00	−209.13	−213.74	−214.03
		^1^*J*(Si,H_α’_)	−273.58	−271.32	−271.49	−275.74	−276.00	−273.58
		^2^*J*(Si,H_β_)	0.88	2.38	1.36	2.15	1.26	1.15
**15**	H_3_Si–CH_3_ Methylsilane	^1^*J*(Si,C)	−54.02	−55.82	−54.77	−58.66	−55.72	−54.93
		^1^*J*(Si,H)	−184.74	−183.56	−183.57	−184.81	−186.05	−184.78
		^2^*J*(Si,H)	8.30	8.96	8.42	9.27	8.59	8.53
**16**	H_3_Si–F Fluorosilane	^1^*J*(Si,H)	−217.11	−216.95	−216.31	−217.40	−218.39	−217.41
		^1^*J*(Si,F)	248.88	257.46	249.12	245.43	247.17	247.66
**17**	SiH_4_ Silane	^1^*J*(Si,H)	−191.02	−190.88	−190.15	−192.1	−192.76	−191.33
**18**	F_3_Si–H Trifluorosilane	^1^*J*(Si,H)	−344.22	−345.76	−344.27	−345.12	−343.74	−343.93
		^1^*J*(Si,F)	239.41	252.97	241.19	243.83	238.43	237.91
**19**	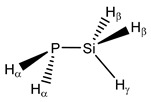 Silylphosphane	^1^*J*(P,Si)	6.82	−5.39	4.72	−3.32	−1.34	4.95
		^1^*J*(P,H_α_)	189.24	186.39	186.98	181.46	187.49	189.96
		^1^*J*(Si,H_β_)	−195.27	−192.96	−193.79	−195.05	−196.09	−195.27
		^1^*J*(Si,H_γ_)	−201.51	−198.33	−199.60	−198.99	−201.44	−201.50
		^2^*J*(P,H_β_)	25.41	24.92	24.84	24.42	25.00	25.27
		^2^*J*(P,H_γ_)	−5.16	−3.44	−4.70	−4.42	−5.17	−5.40
		^2^*J*(Si,H_α_)	8.32	9.44	8.58	10.09	8.87	8.78
**20**	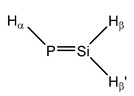 Silylidenephosphane	^1^*J*(P,Si)	125.09	111.02	125.27	110.88	113.64	119.58
		^1^*J*(P,H_α_)	135.77	132.54	133.50	126.41	130.96	136.70
		^1^*J*(Si,H_β_)	−227.93	−223.38	−224.92	−227.49	−227.66	−227.47
		^1^*J*(Si,H_β’_)	−221.37	−219.50	−219.69	−225.64	−223.94	−221.05
		^2^*J*(P,H_β_)	−31.96	−29.86	−31.28	−31.31	−32.09	−32.12
		^2^*J*(P,H_β’_)	36.13	34.66	35.64	35.60	35.42	36.13
		^2^*J*(Si,H_α_)	23.50	24.30	24.24	25.91	24.51	24.57

^1^ SSCCs involving nitrogen were calculated for ^14^N isotope. ^2^ Breaking down the SSCCs calculated with the dyall.aae4z^+^ basis set into four Ramsey’s contributions, FC, SD, PSO, DSO, can be found in Table S2 in the Supporting Information file.

**Table 4:** The sizes of contracted and uncontracted basis sets under consideration.

Basis Set	H	C, N, F	P, Si
pcJ-1/pcJ-1(uc)	10/12	27/34	31/50
pecJ-1/pecJ-1(uc)	11/13	27/35	38/53
pcJ-2/pcJ-2(uc)	24/27	51/62	54/77
pecJ-2/pecJ-2(uc)	20/22	43/51	55/71
aug-cc-pVTZ-J/aug-cc-pVTZ-J(uc)	20/24	46/55	68/87

**Table 5:** Comparison of the theoretical SSCCs calculated with the pecJ-1 and pecJ-2 basis sets with the experimental data (in Hz).

Molecule	SSCC	Basis Set	CCSD					Δ_vib_^1^	Δ_rel_^2^	Δ_sol_^3^	*J*_tot_	*J*_exp_^4^
			*J*_FC_	*J*_SD_	*J*_PSO_	*J*_DSO_	*J*_basic_					
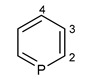 **1**	^1^*J*(P,C_2_)	pecJ-1	−10.14	5.09	−36.72	0.20	−41.57	−4.33	−4.91	2.95	−47.86	(−)53.0
		pecJ-2	−12.23	5.12	−37.5	0.20	−44.41			3.40	−50.25	
	^2^*J*(P,C_3_)	pecJ-1	−13.51	−3.48	0.92	−0.05	−16.12	−1.38	0.00	−0.09	−17.59	−14.0
		pecJ-2	−13.56	−3.42	0.93	−0.05	−16.10			−0.26	−17.74	
	^3^*J*(P,C_4_)	pecJ-1	12.57	8.81	1.67	−0.04	23.01	3.39	−0.24	0.55	26.71	22.0
		pecJ-2	12.21	9.04	1.78	−0.04	22.99			0.68	26.82	
	^2^*J*(P,H_2_)	pecJ-1	42.46	−0.40	−7.07	−0.41	34.58	−0.41	1.47	−0.66	34.98	38.0
		pecJ-2	43.42	−0.3	−7.09	−0.42	35.61			−0.75	35.92	
	^3^*J*(P,H_3_)	pecJ-1	8.71	−0.21	0.65	−0.69	8.46	0.45	−0.45	0.72	9.18	8.0
		pecJ-2	8.58	−0.28	0.70	−0.70	8.30			0.77	9.07	
	^4^*J*(P,H_4_)	pecJ-1	−4.33	−0.29	0.55	−0.64	−4.71	−0.69	0.32	−0.03	−5.11	−3.5
		pecJ-2	−4.17	−0.15	0.61	−0.64	−4.35			−0.07	−4.79	
P≡CH **2**	^1^*J*(P,C)	pecJ-1	19.52	40.96	11.51	0.00	71.99	−5.47	−4.50	−6.66	55.36	56.0
		pecJ-2	19.85	43.26	13.67	0.00	76.78			−5.93	60.88	
	^2^*J*(P,H)	pecJ-1	27.88	3.28	21.48	−1.46	51.18	−4.16	0.24	1.37	48.63	44.0
		pecJ-2	25.73	5.36	23.46	−1.47	53.08			1.17	50.33	
PH_3_ **8**	^1^*J*(P,H)	pecJ-1	179.64	−0.78	5.37	0.03	184.26	−9.34	−2.63	Gas phase	172.29	176.2
		pecJ-2	182.68	−0.91	5.93	0.00	187.70				175.73	
H_3_Si–F **16**	^1^*J*(Si,H)	pecJ-1	−215.64	−0.08	1.14	−0.22	−214.80	−8.82	−3.30	−1.66	−228.58	(−)233.6
		pecJ-2	−213.72	−0.17	1.11	−0.21	−212.99			−1.55	−226.66	
	^1^*J*(Si,F)	pecJ-1	212.82	−7.32	58.57	−0.08	263.99	8.69	10.43	−3.82	279.29	278.7
		pecJ-2	203.39	−7.78	58.88	−0.06	254.43			−3.62	269.93	
SiH_4_ **17**	^1^*J*(Si,H)	pecJ-1	−189.89	−0.02	0.48	−0.03	−189.46	−7.27	−2.96	Gas phase	−199.69	(−)201.9
		pecJ-2	−188.71	−0.07	0.43	−0.02	−188.37				−198.60	

^1^ All zero-point vibrational corrections were calculated using the pecJ-1 basis set. ^2^ All relativistic corrections were calculated using the dyall.acv4z basis set. ^3^ The IEF-PCM model was specified each time for a particular solvent in accordance with the experimental data. ^4^ Experimental values were taken from different sources: **1**—[63], **2**—[64], **8**—[36], **16**—[65], **17**—[66].

## Data Availability

All data are contained within this article or in the Supplementary Materials file.

## Supplementary Materials

The following supporting information can be downloaded at: https://www.mdpi.com/article/10.3390/molecules27196145/s1: Table S1: pecJ-*n* (*n* = 1, 2) basis sets for silicon and phosphorus in Dalton and CFOUR format, equilibrium geometries of all considered molecules; Table S2: SSCCs calculated at the SOPPA(CCSD) level using the dyall.aae4z^+^ basis set with breaking down into four Ramsey’s contributions, FC, SD, PSO, and DSO.
