# Supplementary material for: New pecJ-n (n = 1, 2) Basis Sets for High-Quality Calculations of Indirect Nuclear Spin–Spin Coupling Constants Involving 31P and 29Si: The Advanced PEC Method
Source: Molecules. 2022 Sep 20;27(19):6145. doi: 10.3390/molecules27196145 (PMC9573013; doi:10.3390/molecules27196145)
Supplement: Supplementary file 1 [file molecules-27-06145-s001.zip › molecules-1915649-supplementary.pdf]

molecules

MDPI

Supplementary Information

Article

New pecJ-*n* (*n* = 1, 2) basis sets for high-quality calculations of indirect nuclear spin-spin coupling constants involving ^{31}P and ^{29}Si: the advanced PEC method

Yuriy Yu. Rusakov [1], and Irina L. Rusakova [2,*]

[1] A. E. Favorsky Irkutsk Institute of Chemistry, Siberian Branch of the Russian Academy of Sciences,Favorsky St. 1, 664033 Irkutsk, Russian Federation
[2] A. E. Favorsky Irkutsk Institute of Chemistry, Siberian Branch of the Russian Academy of Sciences,Favorsky St. 1, 664033 Irkutsk, Russian Federation
* Correspondence: i-rusakova@bk.ru

PecJ-1 for silicon (in Dalton format)

```
$ Si
a 14
$ s functions
   14    8    0
   4.247214E+06  1.9707E-06  5.4116E-07  0.00000000  0.00000000  0.00000000  0.00000000  0.00000000  0.00000000
   6.385443E+05  1.9862E-05  5.2659E-06  0.00000000  0.00000000  0.00000000  0.00000000  0.00000000  0.00000000
   7.426145E+04  2.4690E-04  6.2793E-05  0.00000000  0.00000000  0.00000000  0.00000000  0.00000000  0.00000000
   1.232379E+04  1.9047E-03  4.9342E-04  0.00000000  0.00000000  0.00000000  0.00000000  0.00000000  0.00000000
   2.821844E+03  9.9072E-03  2.7064E-03  0.00000000  0.00000000  0.00000000  0.00000000  0.00000000  0.00000000
   7.902749E+02  4.0989E-02  1.1152E-02  0.00000000  0.00000000  0.00000000  0.00000000  0.00000000  0.00000000
   2.530448E+02  1.3225E-01  3.9024E-02  0.00000000  0.00000000  0.00000000  0.00000000  0.00000000  0.00000000
   8.929083E+01  3.0633E-01  1.0033E-01  0.00000000  0.00000000  0.00000000  0.00000000  0.00000000  0.00000000
   3.421840E+01  0.00000000  0.00000000  1.00000000  0.00000000  0.00000000  0.00000000  0.00000000  0.00000000
   1.391189E+01  0.00000000  0.00000000  0.00000000  1.00000000  0.00000000  0.00000000  0.00000000  0.00000000
   4.107635E+00  0.00000000  0.00000000  0.00000000  0.00000000  1.00000000  0.00000000  0.00000000  0.00000000
   1.505666E+00  0.00000000  0.00000000  0.00000000  0.00000000  0.00000000  1.00000000  0.00000000  0.00000000
   2.629620E-01  0.00000000  0.00000000  0.00000000  0.00000000  0.00000000  0.00000000  1.00000000  0.00000000
   1.070097E-01  0.00000000  0.00000000  0.00000000  0.00000000  0.00000000  0.00000000  0.00000000  1.00000000
$ p functions
    8    5    0
   2.945253E+02  4.3124E-03  4.6720E-04  0.00000000  0.00000000  0.00000000
   7.100883E+01  3.1561E-02  3.3501E-03  0.00000000  0.00000000  0.00000000
   2.261134E+01  1.3104E-01  1.5321E-02  0.00000000  0.00000000  0.00000000
   8.243004E+00  3.2485E-01  4.0777E-02  0.00000000  0.00000000  0.00000000
   3.132172E+00  4.5187E-01  5.7443E-02  0.00000000  0.00000000  0.00000000
   1.208189E+00  0.00000000  0.00000000  1.00000000  0.00000000  0.00000000
   3.274731E-01  0.00000000  0.00000000  0.00000000  1.00000000  0.00000000
   7.724492E-02  0.00000000  0.00000000  0.00000000  0.00000000  1.00000000
$ d functions
    3    3    0
   1.278348E+01  1.00000000  0.00000000  0.00000000
   3.601261E+00  0.00000000  1.00000000  0.00000000
   4.850347E-01  0.00000000  0.00000000  1.00000000
```

PecJ-1 for phosphorus (in Dalton format)

```
$ P
a 15
$ s functions
   14    8    0
   4.134637E+06  2.0126E-06  5.4166E-07  0.00000000  0.00000000  0.00000000  0.00000000  0.00000000  0.00000000
   6.209828E+05  2.1498E-05  5.7456E-06  0.00000000  0.00000000  0.00000000  0.00000000  0.00000000  0.00000000
   8.339905E+04  2.6335E-04  6.8919E-05  0.00000000  0.00000000  0.00000000  0.00000000  0.00000000  0.00000000
   1.388436E+04  2.0149E-03  5.5608E-04  0.00000000  0.00000000  0.00000000  0.00000000  0.00000000  0.00000000
   3.197419E+03  1.0496E-02  2.9474E-03  0.00000000  0.00000000  0.00000000  0.00000000  0.00000000  0.00000000
   9.033799E+02  4.2447E-02  1.1981E-02  0.00000000  0.00000000  0.00000000  0.00000000  0.00000000  0.00000000
   2.957004E+02  1.3418E-01  3.9463E-02  0.00000000  0.00000000  0.00000000  0.00000000  0.00000000  0.00000000
   1.063410E+02  3.1480E-01  9.9687E-02  0.00000000  0.00000000  0.00000000  0.00000000  0.00000000  0.00000000
   4.110792E+01  0.00000000  0.00000000  1.00000000  0.00000000  0.00000000  0.00000000  0.00000000  0.00000000
   1.676836E+01  0.00000000  0.00000000  0.00000000  1.00000000  0.00000000  0.00000000  0.00000000  0.00000000
   5.330741E+00  0.00000000  0.00000000  0.00000000  0.00000000  1.00000000  0.00000000  0.00000000  0.00000000
   1.982165E+00  0.00000000  0.00000000  0.00000000  0.00000000  0.00000000  1.00000000  0.00000000  0.00000000
   3.817254E-01  0.00000000  0.00000000  0.00000000  0.00000000  0.00000000  0.00000000  1.00000000  0.00000000
   1.360260E-01  0.00000000  0.00000000  0.00000000  0.00000000  0.00000000  0.00000000  0.00000000  1.00000000
$ p functions
    8    5    0
   3.518121E+02  2.3642E-03  4.2579E-04  0.00000000  0.00000000  0.00000000
   8.341509E+01  1.5226E-02  2.8986E-03  0.00000000  0.00000000  0.00000000
   2.625090E+01  6.2924E-02  1.2752E-02  0.00000000  0.00000000  0.00000000
   9.486400E+00  1.6866E-01  3.2405E-02  0.00000000  0.00000000  0.00000000
   3.620866E+00  2.2882E-01  5.0078E-02  0.00000000  0.00000000  0.00000000
   1.422278E+00  0.00000000  0.00000000  1.00000000  0.00000000  0.00000000
   3.860029E-01  0.00000000  0.00000000  0.00000000  1.00000000  0.00000000
   1.018896E-01  0.00000000  0.00000000  0.00000000  0.00000000  1.00000000
$ d functions
    3    3    0
   1.031033E+01  1.00000000  0.00000000  0.00000000
   2.501292E+00  0.00000000  1.00000000  0.00000000
   4.156433E-01  0.00000000  0.00000000  1.00000000
```

PecJ-2 for silicon (in Dalton format)

```
$ Si
a 14
$ s functions
   17   10    0
   1.999932E+07  1.9318E-07  5.1799E-08  0.00000000  0.00000000  0.00000000  0.00000000  0.00000000  0.00000000  0.00000000  0.00000000
   2.761762E+06  3.5843E-06  9.5989E-07  0.00000000  0.00000000  0.00000000  0.00000000  0.00000000  0.00000000  0.00000000  0.00000000
   2.544618E+05  5.9197E-05  1.5766E-05  0.00000000  0.00000000  0.00000000  0.00000000  0.00000000  0.00000000  0.00000000  0.00000000
   3.835108E+04  4.8610E-04  1.2965E-04  0.00000000  0.00000000  0.00000000  0.00000000  0.00000000  0.00000000  0.00000000  0.00000000
   8.644240E+03  2.6022E-03  6.9449E-04  0.00000000  0.00000000  0.00000000  0.00000000  0.00000000  0.00000000  0.00000000  0.00000000
   2.399797E+03  1.1057E-02  2.9591E-03  0.00000000  0.00000000  0.00000000  0.00000000  0.00000000  0.00000000  0.00000000  0.00000000
   7.843863E+02  3.7612E-02  1.0233E-02  0.00000000  0.00000000  0.00000000  0.00000000  0.00000000  0.00000000  0.00000000  0.00000000
   2.883833E+02  1.0731E-01  3.0417E-02  0.00000000  0.00000000  0.00000000  0.00000000  0.00000000  0.00000000  0.00000000  0.00000000
   1.132075E+02  2.4931E-01  7.8018E-02  0.00000000  0.00000000  0.00000000  0.00000000  0.00000000  0.00000000  0.00000000  0.00000000
   4.651835E+01  0.00000000  0.00000000  1.00000000  0.00000000  0.00000000  0.00000000  0.00000000  0.00000000  0.00000000  0.00000000
   1.979378E+01  0.00000000  0.00000000  0.00000000  1.00000000  0.00000000  0.00000000  0.00000000  0.00000000  0.00000000  0.00000000
   7.836431E+00  0.00000000  0.00000000  0.00000000  0.00000000  1.00000000  0.00000000  0.00000000  0.00000000  0.00000000  0.00000000
   3.345136E+00  0.00000000  0.00000000  0.00000000  0.00000000  0.00000000  1.00000000  0.00000000  0.00000000  0.00000000  0.00000000
   1.371723E+00  0.00000000  0.00000000  0.00000000  0.00000000  0.00000000  0.00000000  1.00000000  0.00000000  0.00000000  0.00000000
   3.643255E-01  0.00000000  0.00000000  0.00000000  0.00000000  0.00000000  0.00000000  0.00000000  1.00000000  0.00000000  0.00000000
   1.746173E-01  0.00000000  0.00000000  0.00000000  0.00000000  0.00000000  0.00000000  0.00000000  0.00000000  1.00000000  0.00000000
   8.206407E-02  0.00000000  0.00000000  0.00000000  0.00000000  0.00000000  0.00000000  0.00000000  0.00000000  0.00000000  1.00000000
$ p functions
    9    6    0
   4.607985E+02  2.0667E-03  2.2055E-04  0.00000000  0.00000000  0.00000000  0.00000000
   1.121558E+02  1.5448E-02  1.7458E-03  0.00000000  0.00000000  0.00000000  0.00000000
```

```
    3.604553E+01   7.2818E-02   7.7727E-03   0.00000000   0.00000000   0.00000000   0.00000000
    1.330447E+01   2.0690E-01   2.4725E-02   0.00000000   0.00000000   0.00000000   0.00000000
    5.219630E+00   3.8471E-01   4.4984E-02   0.00000000   0.00000000   0.00000000   0.00000000
    2.084965E+00   0.00000000   0.00000000   1.00000000   0.00000000   0.00000000   0.00000000
    8.151844E-01   0.00000000   0.00000000   0.00000000   1.00000000   0.00000000   0.00000000
    2.637732E-01   0.00000000   0.00000000   0.00000000   0.00000000   1.00000000   0.00000000
    6.880276E-02   0.00000000   0.00000000   0.00000000   0.00000000   0.00000000   1.00000000
$ d functions
     4    4    0
    1.950340E+01   1.00000000   0.00000000   0.00000000   0.00000000
    5.868198E+00   0.00000000   1.00000000   0.00000000   0.00000000
    1.810024E+00   0.00000000   0.00000000   1.00000000   0.00000000
    3.813274E-01   0.00000000   0.00000000   0.00000000   1.00000000
$ f functions
     1    1    0
    3.510370E-01   1.00000000
```

PecJ-2 for phosphorus (in Dalton format)

```
$ P
a 15
$ s functions
    17   10    0
    1.990072E+07   1.9963E-07   5.3725E-08   0.00000000   0.00000000   0.00000000   0.00000000   0.00000000   0.00000000   0.00000000   0.00000000
    2.800210E+06   4.7298E-06   1.2726E-06   0.00000000   0.00000000   0.00000000   0.00000000   0.00000000   0.00000000   0.00000000   0.00000000
    2.563558E+05   6.2851E-05   1.7050E-05   0.00000000   0.00000000   0.00000000   0.00000000   0.00000000   0.00000000   0.00000000   0.00000000
    4.642895E+04   4.2805E-04   1.1595E-04   0.00000000   0.00000000   0.00000000   0.00000000   0.00000000   0.00000000   0.00000000   0.00000000
    1.081315E+04   2.3250E-03   6.3742E-04   0.00000000   0.00000000   0.00000000   0.00000000   0.00000000   0.00000000   0.00000000   0.00000000
    2.998583E+03   1.0021E-02   2.7619E-03   0.00000000   0.00000000   0.00000000   0.00000000   0.00000000   0.00000000   0.00000000   0.00000000
    9.635223E+02   3.5437E-02   9.9225E-03   0.00000000   0.00000000   0.00000000   0.00000000   0.00000000   0.00000000   0.00000000   0.00000000
    3.449980E+02   1.0533E-01   3.0617E-02   0.00000000   0.00000000   0.00000000   0.00000000   0.00000000   0.00000000   0.00000000   0.00000000
    1.326081E+02   2.4809E-01   7.9408E-02   0.00000000   0.00000000   0.00000000   0.00000000   0.00000000   0.00000000   0.00000000   0.00000000
    5.427039E+01   0.00000000   0.00000000   1.00000000   0.00000000   0.00000000   0.00000000   0.00000000   0.00000000   0.00000000   0.00000000
    2.333299E+01   0.00000000   0.00000000   0.00000000   1.00000000   0.00000000   0.00000000   0.00000000   0.00000000   0.00000000   0.00000000
    9.848763E+00   0.00000000   0.00000000   0.00000000   0.00000000   1.00000000   0.00000000   0.00000000   0.00000000   0.00000000   0.00000000
    4.153065E+00   0.00000000   0.00000000   0.00000000   0.00000000   0.00000000   1.00000000   0.00000000   0.00000000   0.00000000   0.00000000
    1.808222E+00   0.00000000   0.00000000   0.00000000   0.00000000   0.00000000   0.00000000   1.00000000   0.00000000   0.00000000   0.00000000
    9.316385E-01   0.00000000   0.00000000   0.00000000   0.00000000   0.00000000   0.00000000   0.00000000   1.00000000   0.00000000   0.00000000
    2.840944E-01   0.00000000   0.00000000   0.00000000   0.00000000   0.00000000   0.00000000   0.00000000   0.00000000   1.00000000   0.00000000
    8.516245E-02   0.00000000   0.00000000   0.00000000   0.00000000   0.00000000   0.00000000   0.00000000   0.00000000   0.00000000   1.00000000
$ p functions
     9    6    0
    4.670514E+02   2.3769E-03   2.1703E-04   0.00000000   0.00000000   0.00000000   0.00000000
    1.130193E+02   1.8198E-02   1.6850E-03   0.00000000   0.00000000   0.00000000   0.00000000
    3.644899E+01   8.3503E-02   7.9564E-03   0.00000000   0.00000000   0.00000000   0.00000000
    1.354295E+01   2.3749E-01   2.2959E-02   0.00000000   0.00000000   0.00000000   0.00000000
    5.336660E+00   4.2421E-01   4.2858E-02   0.00000000   0.00000000   0.00000000   0.00000000
    2.134318E+00   0.00000000   0.00000000   1.00000000   0.00000000   0.00000000   0.00000000
    7.677971E-01   0.00000000   0.00000000   0.00000000   1.00000000   0.00000000   0.00000000
    2.870326E-01   0.00000000   0.00000000   0.00000000   0.00000000   1.00000000   0.00000000
    9.316817E-02   0.00000000   0.00000000   0.00000000   0.00000000   0.00000000   1.00000000
$ d functions
     4    4    0
    1.910030E+01   1.00000000   0.00000000   0.00000000   0.00000000
    5.559497E+00   0.00000000   1.00000000   0.00000000   0.00000000
    1.328245E+00   0.00000000   0.00000000   1.00000000   0.00000000
    3.484249E-01   0.00000000   0.00000000   0.00000000   1.00000000
$ f functions
     1    1    0
    4.679524E-01   1.00000000
```

PecJ-1 for silicon (in CFOUR format)
SI:pecJ-1
Property-energy consistent basis set

```
  3
    0    1    2
    8    5    3
   14    8    3

4.247214E+06  6.385443E+05  7.426145E+04  1.232379E+04  2.821844E+03
7.902749E+02  2.530448E+02  8.929083E+01  3.421840E+01  1.391189E+01
4.107635E+00  1.505666E+00  2.629620E-01  1.070097E-01

 1.9707E-06  5.4116E-07  0.00000000  0.00000000  0.00000000  0.00000000  0.00000000
 0.00000000
 1.9862E-05  5.2659E-06  0.00000000  0.00000000  0.00000000  0.00000000  0.00000000
 0.00000000
 2.4690E-04  6.2793E-05  0.00000000  0.00000000  0.00000000  0.00000000  0.00000000
 0.00000000
 1.9047E-03  4.9342E-04  0.00000000  0.00000000  0.00000000  0.00000000  0.00000000
 0.00000000
 9.9072E-03  2.7064E-03  0.00000000  0.00000000  0.00000000  0.00000000  0.00000000
 0.00000000
 4.0989E-02  1.1152E-02  0.00000000  0.00000000  0.00000000  0.00000000  0.00000000
 0.00000000
 1.3225E-01  3.9024E-02  0.00000000  0.00000000  0.00000000  0.00000000  0.00000000
 0.00000000
 3.0633E-01  1.0033E-01  0.00000000  0.00000000  0.00000000  0.00000000  0.00000000
 0.00000000
 0.00000000  0.00000000  1.00000000  0.00000000  0.00000000  0.00000000  0.00000000
 0.00000000
 0.00000000  0.00000000  0.00000000  1.00000000  0.00000000  0.00000000  0.00000000
 0.00000000
 0.00000000  0.00000000  0.00000000  0.00000000  1.00000000  0.00000000  0.00000000
 0.00000000
 0.00000000  0.00000000  0.00000000  0.00000000  0.00000000  1.00000000  0.00000000
 0.00000000
 0.00000000  0.00000000  0.00000000  0.00000000  0.00000000  0.00000000  1.00000000
 0.00000000
 0.00000000  0.00000000  0.00000000  0.00000000  0.00000000  0.00000000  0.00000000
 1.00000000

2.945253E+02  7.100883E+01  2.261134E+01  8.243004E+00  3.132172E+00
1.208189E+00  3.274731E-01  7.724492E-02

 4.3124E-03  4.6720E-04  0.00000000  0.00000000  0.00000000
 3.1561E-02  3.3501E-03  0.00000000  0.00000000  0.00000000
 1.3104E-01  1.5321E-02  0.00000000  0.00000000  0.00000000
 3.2485E-01  4.0777E-02  0.00000000  0.00000000  0.00000000
 4.5187E-01  5.7443E-02  0.00000000  0.00000000  0.00000000
 0.00000000  0.00000000  1.00000000  0.00000000  0.00000000
```

0.00000000 0.00000000 0.00000000 1.00000000 0.00000000
0.00000000 0.00000000 0.00000000 0.00000000 1.00000000

1.278348E+01 3.601261E+00 4.850347E-01

1.00000000 0.00000000 0.00000000
0.00000000 1.00000000 0.00000000
0.00000000 0.00000000 1.00000000

PecJ-1 for phosphorus (in CFOUR format)
P:pecJ-1
Property-energy consistent basis set

3
0 1 2
8 5 3
14 8 3

4.134637E+06 6.209828E+05 8.339905E+04 1.388436E+04 3.197419E+03
9.033799E+02 2.957004E+02 1.063410E+02 4.110792E+01 1.676836E+01
5.330741E+00 1.982165E+00 3.817254E-01 1.360260E-01

2.0126E-06 5.4166E-07 0.00000000 0.00000000 0.00000000 0.00000000 0.00000000
0.00000000
2.1498E-05 5.7456E-06 0.00000000 0.00000000 0.00000000 0.00000000 0.00000000
0.00000000
2.6335E-04 6.8919E-05 0.00000000 0.00000000 0.00000000 0.00000000 0.00000000
0.00000000
2.0149E-03 5.5608E-04 0.00000000 0.00000000 0.00000000 0.00000000 0.00000000
0.00000000
1.0496E-02 2.9474E-03 0.00000000 0.00000000 0.00000000 0.00000000 0.00000000
0.00000000
4.2447E-02 1.1981E-02 0.00000000 0.00000000 0.00000000 0.00000000 0.00000000
0.00000000
1.3418E-01 3.9463E-02 0.00000000 0.00000000 0.00000000 0.00000000 0.00000000
0.00000000
3.1480E-01 9.9687E-02 0.00000000 0.00000000 0.00000000 0.00000000 0.00000000
0.00000000
0.00000000 0.00000000 1.00000000 0.00000000 0.00000000 0.00000000 0.00000000
0.00000000
0.00000000 0.00000000 0.00000000 1.00000000 0.00000000 0.00000000 0.00000000
0.00000000
0.00000000 0.00000000 0.00000000 0.00000000 1.00000000 0.00000000 0.00000000
0.00000000
0.00000000 0.00000000 0.00000000 0.00000000 0.00000000 1.00000000 0.00000000
0.00000000
0.00000000 0.00000000 0.00000000 0.00000000 0.00000000 0.00000000 1.00000000
0.00000000
0.00000000 0.00000000 0.00000000 0.00000000 0.00000000 0.00000000 0.00000000
1.00000000

```
3.518121E+02 8.341509E+01 2.625090E+01 9.486400E+00 3.620866E+00
1.422278E+00 3.860029E-01 1.018896E-01

 2.3642E-03 4.2579E-04 0.00000000 0.00000000 0.00000000
 1.5226E-02 2.8986E-03 0.00000000 0.00000000 0.00000000
 6.2924E-02 1.2752E-02 0.00000000 0.00000000 0.00000000
 1.6866E-01 3.2405E-02 0.00000000 0.00000000 0.00000000
 2.2882E-01 5.0078E-02 0.00000000 0.00000000 0.00000000
 0.00000000 0.00000000 1.00000000 0.00000000 0.00000000
 0.00000000 0.00000000 0.00000000 1.00000000 0.00000000
 0.00000000 0.00000000 0.00000000 0.00000000 1.00000000

1.031033E+01 2.501292E+00 4.156433E-01

 1.00000000 0.00000000 0.00000000
 0.00000000 1.00000000 0.00000000
 0.00000000 0.00000000 1.00000000
```

PecJ-2 for silicon (in CFOUR format)

```
SI:pecJ-2
Property-energy consistent basis set

  4
    0    1    2    3
   10    6    4    1
   17    9    4    1

1.999932E+07 2.761762E+06 2.544618E+05 3.835108E+04 8.644240E+03
2.399797E+03 7.843863E+02 2.883833E+02 1.132075E+02 4.651835E+01
1.979378E+01 7.836431E+00 3.345136E+00 1.371723E+00 3.643255E-01
1.746173E-01 8.206407E-02

 1.9318E-07 5.1799E-08 0.00000000 0.00000000 0.00000000 0.00000000 0.00000000
 0.00000000 0.00000000 0.00000000
 3.5843E-06 9.5989E-07 0.00000000 0.00000000 0.00000000 0.00000000 0.00000000
 0.00000000 0.00000000 0.00000000
 5.9197E-05 1.5766E-05 0.00000000 0.00000000 0.00000000 0.00000000 0.00000000
 0.00000000 0.00000000 0.00000000
 4.8610E-04 1.2965E-04 0.00000000 0.00000000 0.00000000 0.00000000 0.00000000
 0.00000000 0.00000000 0.00000000
 2.6022E-03 6.9449E-04 0.00000000 0.00000000 0.00000000 0.00000000 0.00000000
 0.00000000 0.00000000 0.00000000
 1.1057E-02 2.9591E-03 0.00000000 0.00000000 0.00000000 0.00000000 0.00000000
 0.00000000 0.00000000 0.00000000
 3.7612E-02 1.0233E-02 0.00000000 0.00000000 0.00000000 0.00000000 0.00000000
 0.00000000 0.00000000 0.00000000
 1.0731E-01 3.0417E-02 0.00000000 0.00000000 0.00000000 0.00000000 0.00000000
 0.00000000 0.00000000 0.00000000
 2.4931E-01 7.8018E-02 0.00000000 0.00000000 0.00000000 0.00000000 0.00000000
 0.00000000 0.00000000 0.00000000
 0.00000000 0.00000000 1.00000000 0.00000000 0.00000000 0.00000000 0.00000000
```

```
 0.00000000  0.00000000  0.00000000
 0.00000000  0.00000000  0.00000000  1.00000000  0.00000000  0.00000000  0.00000000
 0.00000000  0.00000000  0.00000000
 0.00000000  0.00000000  0.00000000  0.00000000  1.00000000  0.00000000  0.00000000
 0.00000000  0.00000000  0.00000000
 0.00000000  0.00000000  0.00000000  0.00000000  0.00000000  1.00000000  0.00000000
 0.00000000  0.00000000  0.00000000
 0.00000000  0.00000000  0.00000000  0.00000000  0.00000000  0.00000000  1.00000000
 0.00000000  0.00000000  0.00000000
 0.00000000  0.00000000  0.00000000  0.00000000  0.00000000  0.00000000  0.00000000
 1.00000000  0.00000000  0.00000000
 0.00000000  0.00000000  0.00000000  0.00000000  0.00000000  0.00000000  0.00000000
 0.00000000  1.00000000  0.00000000
 0.00000000  0.00000000  0.00000000  0.00000000  0.00000000  0.00000000  0.00000000
 0.00000000  0.00000000  1.00000000

4.607985E+02  1.121558E+02  3.604553E+01  1.330447E+01  5.219630E+00
2.084965E+00  8.151844E-01  2.637732E-01  6.880276E-02

 2.0667E-03  2.2055E-04  0.00000000  0.00000000  0.00000000  0.00000000
 1.5448E-02  1.7458E-03  0.00000000  0.00000000  0.00000000  0.00000000
 7.2818E-02  7.7727E-03  0.00000000  0.00000000  0.00000000  0.00000000
 2.0690E-01  2.4725E-02  0.00000000  0.00000000  0.00000000  0.00000000
 3.8471E-01  4.4984E-02  0.00000000  0.00000000  0.00000000  0.00000000
 0.00000000  0.00000000  1.00000000  0.00000000  0.00000000  0.00000000
 0.00000000  0.00000000  0.00000000  1.00000000  0.00000000  0.00000000
 0.00000000  0.00000000  0.00000000  0.00000000  1.00000000  0.00000000
 0.00000000  0.00000000  0.00000000  0.00000000  0.00000000  1.00000000

1.950340E+01  5.868198E+00  1.810024E+00  3.813274E-01

 1.00000000  0.00000000  0.00000000  0.00000000
 0.00000000  1.00000000  0.00000000  0.00000000
 0.00000000  0.00000000  1.00000000  0.00000000
 0.00000000  0.00000000  0.00000000  1.00000000

3.510370E-01

 1.00000000
```

PecJ-2 for phosphorus (in CFOUR format)
P:pecJ-2
Property-energy consistent basis set

```
 4
   0   1   2   3
  10   6   4   1
  17   9   4   1

1.990072E+07  2.800210E+06  2.563558E+05  4.642895E+04  1.081315E+04
2.998583E+03  9.635223E+02  3.449980E+02  1.326081E+02  5.427039E+01
```

2.333299E+01 9.848763E+00 4.153065E+00 1.808222E+00 9.316385E-01
2.840944E-01 8.516245E-02

1.9963E-07 5.3725E-08 0.00000000 0.00000000 0.00000000 0.00000000 0.00000000
0.00000000 0.00000000 0.00000000
4.7298E-06 1.2726E-06 0.00000000 0.00000000 0.00000000 0.00000000 0.00000000
0.00000000 0.00000000 0.00000000
6.2851E-05 1.7050E-05 0.00000000 0.00000000 0.00000000 0.00000000 0.00000000
0.00000000 0.00000000 0.00000000
4.2805E-04 1.1595E-04 0.00000000 0.00000000 0.00000000 0.00000000 0.00000000
0.00000000 0.00000000 0.00000000
2.3250E-03 6.3742E-04 0.00000000 0.00000000 0.00000000 0.00000000 0.00000000
0.00000000 0.00000000 0.00000000
1.0021E-02 2.7619E-03 0.00000000 0.00000000 0.00000000 0.00000000 0.00000000
0.00000000 0.00000000 0.00000000
3.5437E-02 9.9225E-03 0.00000000 0.00000000 0.00000000 0.00000000 0.00000000
0.00000000 0.00000000 0.00000000
1.0533E-01 3.0617E-02 0.00000000 0.00000000 0.00000000 0.00000000 0.00000000
0.00000000 0.00000000 0.00000000
2.4809E-01 7.9408E-02 0.00000000 0.00000000 0.00000000 0.00000000 0.00000000
0.00000000 0.00000000 0.00000000
0.00000000 0.00000000 1.00000000 0.00000000 0.00000000 0.00000000 0.00000000
0.00000000 0.00000000 0.00000000
0.00000000 0.00000000 0.00000000 1.00000000 0.00000000 0.00000000 0.00000000
0.00000000 0.00000000 0.00000000
0.00000000 0.00000000 0.00000000 0.00000000 1.00000000 0.00000000 0.00000000
0.00000000 0.00000000 0.00000000
0.00000000 0.00000000 0.00000000 0.00000000 0.00000000 1.00000000 0.00000000
0.00000000 0.00000000 0.00000000
0.00000000 0.00000000 0.00000000 0.00000000 0.00000000 0.00000000 1.00000000
0.00000000 0.00000000 0.00000000
0.00000000 0.00000000 0.00000000 0.00000000 0.00000000 0.00000000 0.00000000
1.00000000 0.00000000 0.00000000
0.00000000 0.00000000 0.00000000 0.00000000 0.00000000 0.00000000 0.00000000
0.00000000 1.00000000 0.00000000
0.00000000 0.00000000 0.00000000 0.00000000 0.00000000 0.00000000 0.00000000
0.00000000 0.00000000 1.00000000

4.670514E+02 1.130193E+02 3.644899E+01 1.354295E+01 5.336660E+00
2.134318E+00 7.677971E-01 2.870326E-01 9.316817E-02

2.3769E-03 2.1703E-04 0.00000000 0.00000000 0.00000000 0.00000000
1.8198E-02 1.6850E-03 0.00000000 0.00000000 0.00000000 0.00000000
8.3503E-02 7.9564E-03 0.00000000 0.00000000 0.00000000 0.00000000
2.3749E-01 2.2959E-02 0.00000000 0.00000000 0.00000000 0.00000000
4.2421E-01 4.2858E-02 0.00000000 0.00000000 0.00000000 0.00000000
0.00000000 0.00000000 1.00000000 0.00000000 0.00000000 0.00000000
0.00000000 0.00000000 0.00000000 1.00000000 0.00000000 0.00000000
0.00000000 0.00000000 0.00000000 0.00000000 1.00000000 0.00000000
0.00000000 0.00000000 0.00000000 0.00000000 0.00000000 1.00000000

1.910030E+01 5.559497E+00 1.328245E+00 3.484249E-01

1.00000000 0.00000000 0.00000000 0.00000000
0.00000000 1.00000000 0.00000000 0.00000000
0.00000000 0.00000000 1.00000000 0.00000000
0.00000000 0.00000000 0.00000000 1.00000000

4.679524E-01

1.00000000

Table S1. Equilibrium geometries of all molecules considered in the work

All geometry optimizations were performed using the CCSD method without taking into account media effects (gas phase). For our fitting molecules (PH_3, HCP, SiH_4, and HSiCH) we have used the aug-cc-pV5Z basis set on all atoms, for the rest of molecules, we have used the aug-cc-pVQZ basis on all atoms.

#	Molecule	Equilibrium XYZ coordinates, Å			
**1**	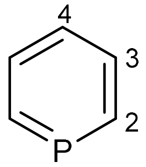 phosphabenzene	P	0.000000	0.000000	1.477237
		C	0.000000	-1.327876	0.367615
		C	0.000000	-1.217181	-1.011615
		C	0.000000	0.000000	-1.676779
		C	0.000000	1.217181	-1.011615
		C	0.000000	1.327876	0.367615
		H	0.000000	-2.317240	0.794297
		H	0.000000	-2.118368	-1.602785
		H	0.000000	0.000000	-2.752908
		H	0.000000	2.118368	-1.602785
		H	0.000000	2.317240	0.794297
**2**	P≡CH phosphaethyne	P	0.000000	0.000000	1.531154
		C	0.000000	0.000000	0.000000
		H	0.000000	0.000000	-1.066380
**3**	$H_2N{-}PH_2$ phosphanamine	P	0.034607	0.621248	0.000000
		N	0.034607	-1.134252	0.000000
		H	0.549303	-1.467631	-0.806618
		H	0.549303	-1.467631	0.806618
		H	-0.929975	0.778149	1.019489
		H	-0.929975	0.778149	-1.019489
**4**	$O{=}PH_3$ phosphine oxide	P	0.000000	0.000000	0.379363
		O	0.000000	0.000000	-1.089860
		H	0.000000	1.250663	1.009480
		H	-1.083106	-0.625332	1.009480
		H	1.083106	-0.625332	1.009480

**5**	PF_3 Phosphorus trifluoride	P	0.000000	0.000000	0.497982
		F	0.000000	1.354565	-0.276656
		F	1.173087	-0.677282	-0.276656
		F	-1.173087	-0.677282	-0.276656
**6**	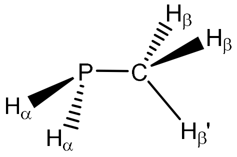 methylphosphane	P	0.069633	0.668757	0.000000
		C	0.069633	-1.180545	0.000000
		H	-0.875503	0.861429	1.027155
		H	-0.875503	0.861429	-1.027155
		H	0.606672	-1.530103	-0.876303
		H	0.606672	-1.530103	0.876303
		H	-0.924629	-1.610730	0.000000
**7**	$H_2P{-}F$ fluorophosphane	P	0.073704	0.538726	0.000000
		F	0.073704	-1.060170	0.000000
		H	-0.884450	0.730321	1.017671
		H	-0.884450	0.730321	-1.017671
**8**	PH_3 phosphane	P	0.000000	0.000000	0.000000
		H	0.000000	0.000000	1.407506
		H	1.405004	0.000000	-0.083876
		H	-0.089032	-1.402180	-0.083876
**9**	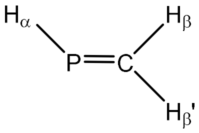 methylenephosphane	P	-0.056176	0.593051	0.000000
		C	-0.056176	-1.066980	0.000000
		H	1.346678	0.781636	0.000000
		H	0.830128	-1.679910	0.000000
		H	-0.997119	-1.595608	0.000000
**10**	$F_2P{-}H$ difluorophosphane	P	-0.041147	0.536958	0.000000
		F	-0.041147	-0.489448	1.198219
		F	-0.041147	-0.489448	-1.198219
		H	1.357849	0.755709	0.000000
**11**	$H_2C{=}SiH_2$ methylenesilane	Si	0.000000	0.000000	0.547212
		C	0.000000	0.000000	-1.147846
		H	0.000000	-1.227851	1.335646
		H	0.000000	1.227851	1.335646
		H	0.000000	0.911979	-1.722593
		H	0.000000	-0.911979	-1.722593
**12**	$CH{\equiv}SiH$ methylidynesilane	Si	0.000000	0.000000	0.000000
		C	0.000000	0.000000	1.580713
		H	0.000000	0.000000	-1.286505
		H	0.000000	0.000000	2.867218
**13**	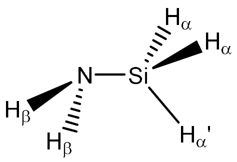 silanamine	Si	0.016426	0.572291	0.000000
		N	0.016426	-1.138484	0.000000
		H	-0.235256	-1.649977	0.823482
		H	-0.235256	-1.649977	-0.823482
		H	0.718863	1.024007	-1.206112
		H	-1.312151	1.209258	0.000000
		H	0.718863	1.024007	1.206112
**14**	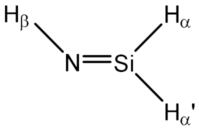 silanimine	Si	0.028991	0.483676	0.000000
		N	0.028991	-1.102111	0.000000
		H	-0.814551	-1.648650	0.000000
		H	-1.105564	1.415596	0.000000
		H	1.311309	1.176368	0.000000

**15**	H_3Si-CH_3 methylsilane	Si	0.000000	0.000000	0.630591
		C	0.000000	0.000000	-1.233695
		H	0.000000	-1.014793	-1.622656
		H	-0.878836	0.507396	-1.622656
		H	0.878836	0.507396	-1.622656
		H	0.000000	1.375412	1.147287
		H	-1.191142	-0.687706	1.147287
		H	1.191142	-0.687706	1.147287
**16**	H_3Si-F fluorosilane	Si	0.000000	0.000000	0.497164
		F	0.000000	0.000000	-1.092817
		H	0.000000	1.388167	0.958353
		H	-1.202188	-0.694083	0.958353
		H	1.202188	-0.694083	0.958353
**17**	SiH_4 silane	Si	0.00000000	0.00000000	0.00000000
		H	0.00000000	-2.26234867	1.59972209
		H	0.00000000	2.26234867	1.59972209
		H	2.26234867	0.00000000	-1.59972209
		H	-2.26234867	0.00000000	-1.59972209
**18**	F_3Si-H trifluorosilane	Si	0.000000	0.000000	0.320657
		F	0.000000	1.461894	-0.231575
		F	-1.266038	-0.730947	-0.231575
		F	1.266038	-0.730947	-0.231575
		H	0.000000	0.000000	1.763333
**19**	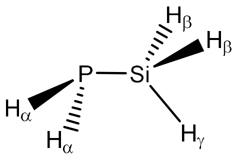 silylphosphane	P	0.053513	1.164365	0.000000
		Si	0.053513	-1.089360	0.000000
		H	-0.901433	1.272827	-1.031856
		H	-0.901433	1.272827	1.031856
		H	0.772326	-1.544725	1.195271
		H	-1.293648	-1.670636	0.000000
		H	0.772326	-1.544725	-1.195271
**20**	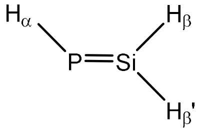 silylidenephosphane	P	-0.042412	1.082238	0.000000
		Si	-0.042412	-0.979393	0.000000
		H	1.372878	1.077309	0.000000
		H	1.139505	-1.843405	0.000000
		H	-1.282444	-1.755982	0.000000

Table S2. SSCCs (in Hz) calculated at the SOPPA(CCSD) level using dyall.aae4z$^+$ basis set with breaking down into four Ramsey's contributions, FC, SD, PSO, DSO.

#	Molecule	SSCC	FC	SD	PSO	DSO	Total *J*
**1**	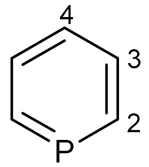 phosphabenzene	$^1J(P,C_2)$	-11.92	6.67	-41.22	0.20	-46.27
		$^2J(P,C_3)$	-16.48	-4.60	1.25	-0.05	-19.88
		$^3J(P,C_4)$	14.58	12.69	2.29	-0.04	29.52
		$^2J(P,H_2)$	41.53	-0.20	-7.95	-0.42	32.96
		$^3J(P,H_3)$	10.19	-0.40	0.40	-0.70	9.49
		$^4J(P,H_4)$	-6.51	-0.08	0.23	-0.65	-7.01
**2**	P≡CH phosphaethyne	$^1J(P,C)$	17.12	48.12	12.88	-0.01	78.11
		$^2J(P,H)$	24.68	5.85	24.66	-1.47	53.72
**3**	H_2N-PH_2 phosphanamine	$^1J(P,N)$	-9.52	3.81	1.96	0.02	-3.73
		$^1J(P,H)$	191.67	-1.34	0.76	0.25	191.34
		$^2J(P,H)$	9.76	0.55	2.19	-0.69	11.81
**4**	$O=PH_3$ phosphine oxide	$^1J(P,H)$	445.16	-0.30	-0.28	0.47	445.05
**5**	PF_3 Phosphorus trifluoride	$^1J(P,F)$	-1183.81	34.09	-260.66	0.81	-1409.57
**6**	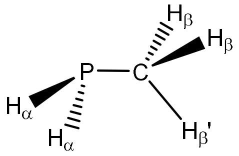 methylphosphane	$^1J(P,C)$	-8.34	7.06	1.20	0.08	0.00
		$^1J(P,H_\alpha)$	192.08	-1.18	4.09	0.20	195.19
		$^2J(P,H_\beta)$	6.85	0.40	0.74	-0.43	7.56
		$^2J(P,H_{\beta'})$	-7.62	-0.31	-0.35	-0.53	-8.81
**7**	H_2P-F fluorophosphane	$^1J(P,F)$	-941.85	92.41	51.22	0.13	-798.09
		$^1J(P,H)$	195.43	-1.48	-2.73	0.37	191.59
**8**	PH_3 phosphane	$^1J(P,H)$	186.19	-1.10	5.55	-0.01	190.63
**9**	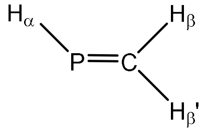 methylenephosphane	$^1J(P,C)$	18.29	18.26	-62.15	0.04	-25.56
		$^1J(P,H_\alpha)$	132.72	-2.62	1.36	0.17	131.63
		$^2J(P,H_\beta)$	-22.64	0.16	-9.75	-0.88	-33.11
		$^2J(P,H_{\beta'})$	36.92	-0.20	-11.77	-0.77	24.18
**10**	F_2P-H difluorophosphane	$^1J(P,H)$	206.23	-1.14	-4.37	0.80	201.52
		$^1J(P,F)$	-1047.29	50.65	-156.74	0.45	-1152.93
**11**	$H_2C=SiH_2$ methylenesilane	$^1J(Si,C)$	-137.29	-6.16	13.40	-0.02	-130.07
		$^1J(Si,H)$	-242.61	0.17	0.67	-0.07	-241.84
		$^2J(Si,H)$	1.92	0.17	2.38	0.38	4.85
**12**	CH≡SiH methylidynesilane	$^1J(Si,C)$	-336.42	-21.34	-21.08	-0.01	-378.85
		$^1J(Si,H)$	-451.07	1.18	3.99	0.00	-445.90
		$^2J(Si,H)$	-106.56	-0.19	-5.72	0.47	-112.00

**13**	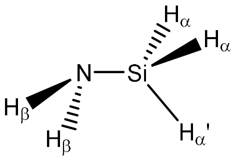 silanamine	^1J(Si,N)	-16.49	-0.25	1.49	-0.01	-15.26
		^1J(Si,H$_\alpha$)	-199.63	-0.07	0.76	-0.15	-199.09
		^1J(Si,H$_{\alpha'}$)	-189.44	-0.06	0.80	-0.15	-188.85
		^2J(Si,H$_\beta$)	-1.68	-0.06	0.15	0.48	-1.11
**14**	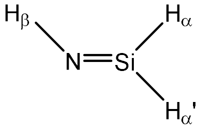 silanimine	^1J(Si,N)	-9.21	-1.74	6.42	0.00	-4.53
		^1J(Si,H$_\alpha$)	-215.67	-0.10	1.16	-0.10	-214.71
		^1J(Si,H$_{\alpha'}$)	-274.41	-0.08	1.02	-0.11	-273.58
		^2J(Si,H$_\beta$)	-3.02	0.23	3.07	0.60	0.88
**15**	$H_3Si{-}CH_3$ methylsilane	^1J(Si,C)	-54.03	-1.45	1.50	-0.04	-54.02
		^1J(Si,H)	-185.14	-0.03	0.54	-0.11	-184.74
		^2J(Si,H)	8.25	-0.18	0.01	0.22	8.30
**16**	$H_3Si{-}F$ fluorosilane	^1J(Si,H)	-217.86	-0.16	1.12	-0.21	-217.11
		^1J(Si,F)	199.87	-7.98	57.04	-0.05	248.88
**17**	SiH_4 silane	^1J(Si,H)	-191.37	-0.05	0.42	-0.02	-191.02
**18**	$F_3Si{-}H$ trifluorosilane	^1J(Si,H)	-344.66	-0.38	1.44	-0.62	-344.22
		^1J(Si,F)	166.41	-4.79	78.18	-0.39	239.41
**19**	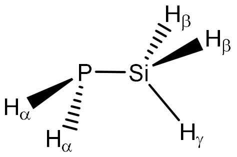 silylphosphane	^1J(P,Si)	11.69	-9.77	4.93	-0.03	6.82
		^1J(P,H$_\alpha$)	182.99	-0.72	6.71	0.26	189.24
		^1J(Si,H$_\beta$)	-195.65	-0.08	0.60	-0.14	-195.27
		^1J(Si,H$_\gamma$)	-201.91	-0.10	0.63	-0.13	-201.51
		^2J(P,H$_\beta$)	25.39	0.06	0.32	-0.36	25.41
		^2J(P,H$_\gamma$)	-4.48	-0.22	0.01	-0.47	-5.16
		^2J(Si,H$_\alpha$)	8.29	-0.11	0.06	0.08	8.32
**20**	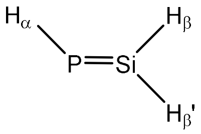 silylidenephosphane	^1J(P,Si)	64.89	-23.28	83.49	-0.01	125.09
		^1J(P,H$_\alpha$)	130.54	-1.19	6.13	0.29	135.77
		^1J(Si,H$_\beta$)	-229.25	0.06	1.36	-0.10	-227.93
		^1J(Si,H$_{\beta'}$)	-222.63	0.09	1.28	-0.11	-221.37
		^2J(P,H$_\beta$)	-27.40	0.40	-4.18	-0.78	-31.96
		^2J(P,H$_{\beta'}$)	40.23	0.51	-3.92	-0.69	36.13
		^2J(Si,H$_\alpha$)	20.80	-0.07	2.71	0.06	23.50
